# Inter-day reliability of surface electromyography recordings of the lumbar part of erector spinae longissimus and trapezius descendens during box lifting

**DOI:** 10.1186/s12891-017-1872-y

**Published:** 2017-12-11

**Authors:** Mikkel Brandt, Lars Louis Andersen, Afshin Samani, Markus Due Jakobsen, Pascal Madeleine

**Affiliations:** 1 National Research Centre for the Working Environment, Lersø Parkalle 105, 2100 Copenhagen, Denmark; 2 Physical Activity and Human Performance group - SMI, Department of Health Science and Technology, Aalborg University, Fredrik Bajers Vej 7, 9220 Aalborg, Denmark

**Keywords:** Working environment, Low back pain, Musculoskeletal pain, Musculoskeletal disorders, Occupational injuries, Shoulder pain

## Abstract

**Background:**

Low back pain and neck-shoulder pain are the most reported types of work-related musculoskeletal disorders, and performing heavy lifting at work and working with trunk rotation increase the risk of developing work-related musculoskeletal disorders. Surface electromyography (sEMG) provides information about the electrical activity of muscles. Thus it has the potential to retrieve indirect information about the physical exposure of specific muscles of workers during their actual work. This study aimed to investigate the inter-day reliability of absolute and normalized amplitude of sEMG measurements obtained during repeated standardized reference lifts.

**Methods:**

The inter-day reliability of sEMG of the erector spinae longissimus and trapezius descendens muscles was tested during standardized box lifts. The lifts were performed with loads of 3, 15 and 30 kg from floor to table and from table to table in three conditions, i.e., forearm length (short reaching distance), ¾ arm length (long reaching distance) and forearm length with trunk rotation. Absolute and normalized root mean square (absRMS and normRMS) values were extracted. In line with the guidelines for reporting reliability and agreement studies, we reported relative and absolute reliability estimated by intra class correlation (ICC_3,K_), standard error of measurement (SEM) and minimal detectable change in percent (MDC).

**Results:**

The ICC_3,K_ was higher for absRMS compared with normRMS while SEM and maximal voluntary contraction (MVC) were similar. A total of 50 out of 56, i.e., 89%, and 41 out of 56, i.e., 73%, of the lifting situations were in the range from moderate to almost perfect for absRMS and normRMS, respectively. The SEM and MDC shoved more variation in the lifting situations performed from floor to table and in the trapezius descendens muscle than in the erector spinae longissimus muscle.

**Conclusion:**

This reliability study showed that maximum absRMS and normRMS were found to have a fair to substantial relative inter-day reliability for most lifts but were more reliable when lifting from table to table than from floor to table for both trapezius descendens and erector spinae muscles. The relative inter-day reliability was higher for absolute compared with normalized sEMG amplitudes while the absolute reliability was similar.

## Background

Low back pain and neck-shoulder pain are the most reported types of work-related musculoskeletal disorders in the construction industry [1] and are associated with decreased work ability, long-term sickness absence and premature withdrawal from working life [2–5]. In a systematic review, Mayer et al. (2012) reported that manual material handling, vibration, trunk flexion or trunk rotation and working with hands above shoulder level increase complaints of the neck and shoulders [6]. Moreover, performing heavy lifting at work and working with trunk rotation increase the risk of developing work-related musculoskeletal disorders [7]. Further, working with trunk flexion, trunk rotation or lifting heavy workloads increases the risk of long-term sickness absence [8]. Especially, construction workers are exposed to a high level of heavy lifting and are frequently working with trunk rotation and elevated arms [8–10].

During recent years, the technological development has allowed measurements outside the laboratory environment of physical exposure (defined as excessive physical load) during the actual working day [11–13]. Surface electromyography (sEMG) provides information about the electrical activity of muscles. sEMG can be used to assess the physical exposure of superficial muscles of workers performing their actual work [11, 13]. Our research group is currently conducting an intervention study aiming at decreasing physical exposure through participatory workshops based on the participants’ physical exposure. In this study, sEMG and accelerometer measurements and video recordings are conducted simultaneously [14]. In this randomized controlled trial, biomechanical events with high physical exposure will be detected. For that purpose, sEMG events during heavy lifting above the sEMG threshold recorded during standardized reference lifts will be recorded from an entire working day and stored for participatory workshops. A crucial point of such methods with repeated measures is the reliability of the sEMG signal across days. As field-based sEMG measurements have become more frequent, understanding the inter-day reliability of normalized sEMG data is critical. The reliability of outcomes should be addressed by relative indices of reliability such as intra class correlation coefficient (ICC) as well as absolute indices of reliability such as standard error of measurement (SEM) and minimal detectable change (MDC) [15]. Previous studies have tested the reliability of sEMG for the trapezius descendens (trapezius) and the lumbar part of erector spinae longissimus (erector spinae) muscles (Table 1) with promising results. However, the reliability of sEMG from these muscles during standardized lifting has not been fully examined.

**Table 1:** Description and results from previous reliability studies

Study	n	Test positions	Body segment - muscle	Movement	Reliability
Schinkel-Ivy et al. (2015)	30 with no history of back pain	Maximum flexion	Back - Lumbar erector spinae (left and Right)	Flexion	ICC = 0.93-0.95, SEM (%) = 9.9
	(15 female and 15 males)	Maximum axial twist	Back - Lumbar erector spinae (left and Right)	Twisting	ICC = 0.87-0.92, SEM (%) = 17.3-20.3
		Maximum flexion	Back - Lower-thoracic erector spinae (left and Right)	Flexion	ICC = 0.84-0.89, SEM (%) = 19.3-19.5
		Maximum axial twist	Back - Lower-thoracic erector spinae (left and Right)	Twisting	ICC = 0.82-0.93, SEM(%) = 17.6-24
Andersen KS et al. (2014)	24 healthy	Arm flexion (45°)	Shoulder - Trapezius superior	Isometric	ICC absEMG = 0.88, ICC nEMG = 0.72
	(14 women and 10 men)	Arm flexion (90°)	Shoulder - Trapezius superior	Isometric	ICC absEMG = 0.82, ICC nEMG = 0.60
		Arm abduction	Shoulder - Trapezius superior	Isometric	ICC absEMG = 0.90, ICC nEMG = 0.72
		Arm flexion	Shoulder - Trapezius superior	Dynamic	ICC absEMG = 0.99, ICC nEMG = 0.96
Michener et al. (2016)	12 with shoulder pain	Scapular plane elevation	Sholuder - Upper trapezius	Ascending phase (30°-120°) (% ref)	ICC = 0.53, SEM = 21.4, MDC = 30.3
	(8 male and 4 women)	Scapular plane elevation	Sholuder - Upper trapezius	Descending phase (30°-120°) (% ref)	ICC = 0.81, SEM = 7.0, MDC = 10.3
Ghofrani et al. (2017)	7	Box lifting from floor to table	Back - Erector spinae (left and right)	Dynamic	ICC = 0.45-0.83, SEM = 34.22-119.22
	(Males)				
Seitz & Uhl (2012)	16 with no shoulder pain	Overhead lifting task	Shoulder - Upper trapezius	Concentric phase (%MVIC)	Intra-session; ICC = 0.98, SEM = 1.7, MDC = 2.5
					Inter-session; ICC = 0.66, SEM = 8.0, MDC = 11.4
	(8 females and 8 males)	Overhead lifting task	Shoulder - Upper trapezius	Eccentric phase (%MVIC)	Intra-session; ICC = 0.96, SEM = 1.3, MDC = 1.9
					Inter-session; ICC = 0.78, SEM = 3.5, MDC = 4.9

The present study aimed to investigate the inter-day reliability of the absolute and normalized amplitude of sEMG measurements obtained during repeated standardized reference lifts. For that purpose, we conducted a study among healthy male subjects testing the inter-day reliability of absolute and normalized root mean square (RMS) values of sEMG recordings during standardized reference lifts in a laboratory environment. The presentation of this reliability study follows the guidelines for reporting reliability and agreement studies (GRRAS) [16]. Of note, we extracted the maximal amplitude of the sEMG during standardized lifts to address the reliability during maximum muscular load.

## Methods

### Participants

Twenty healthy male participants volunteered to participate in the study at Aalborg University, Denmark. Three participants were excluded due to technical problems (electrodes, noise) with the sEMG equipment. Table 2 presents anthropometric information for the remaining seventeen participants. Inclusion criteria were healthy males aged 18-60 years, and exclusion criteria were blood pressure above 160/100 mmHg, life-threatening diseases (e.g. ischemic heart disease, previous stroke), herniated disc and current or previous injuries (within the last 12 months) in the back or shoulder regions.

**Table 2:** Anthropometric data for the study population presented as mean ± SD

Anthropometric data	
N	17
Age (yr)	28.6 ± 10.0
Height (cm)	179.4 ± 7.1
Body mass (kg)	76.4 ± 10.0
BMI (kg/m2)	23.8 ± 2.7
Dom hand (R/L)	R (16) / L (1)

### Ethics, consent and permissions

In accordance with the Helsinki Declaration, all participants were informed about the objective and the procedures of the study before providing written informed consent to participate. The study was approved by the North Denmark Region Committee on Health Research (N-20160023).

### Study protocol

All participants attended two sessions with an interval of 13.8 ± 1.1 days to test the inter-day reliability of sEMG measurements during lifting tasks. Prior to and after the lifting tasks, the subjects performed three bilateral isometric MVCs for the trapezius and erector spinae muscles with 1-2 min of rest in between. For the trapezius muscle, the subject performed 90° shoulder abduction against static resistance from the test leader. For the erector spinae muscle, the subjects lay prone with the nose facing the floor on a customized back extension apparatus supporting the subjects’ legs and raised the body from the floor [17]. The subjects performed back extensions from a position with a slightly flexed back and pushed, at the level of C7 on the back, against a static resistance applied by the test leader.

**Fig. 1:**
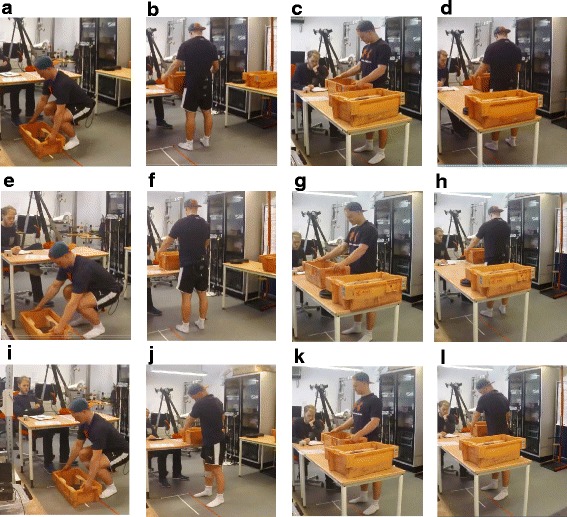
Illustration of the lifting protocol. **a** Lift from floor to table with forearm length (horizontal distance of the load center to the body) – starting position. **b** Lift from floor to table with forearm length – end position. **c** Lift from table to table with forearm length – starting position. **d** Lift from table to table with forearm length – end position. **e** Lift from floor to table with ¾ forearm length (horizontal distance of the load center to the body) – starting position. **f** Lift from floor to table with ¾ forearm length – end position. **g** Lift from table to table with ¾ forearm length – starting position. **h** Lift from table to table with ¾ forearm length – end position. **i** Lift from floor to table with rotation of the trunk – starting position. **j** Lift from floor to table with rotation of the trunk – end position. **k** Lift from table to table with rotation of the trunk – starting position. **l** Lift from table to table with rotation of the trunk – end position. See methods section for more details

The subjects lifted a box (W: 56 cm, L: 34 cm, H: 20 cm (Fig. 1)) with a load of either 3, 15 or 30 kg from the floor to a table (height 72 cm) and from one table to another in three conditions, i.e. forearm length (short reaching distance), ¾ arm length (long reaching distance) and forearm length with trunk rotation (trunk rotation). The lifting conditions are described in detail below. The subjects were instructed to lift the box with their preferred lifting strategy in a slow controlled manner (~2-4 s) and were to start the lifts at the test leader’s signal. The recording was initiated 2 seconds prior to the start of the lift and terminated 2 seconds after the lift. During this time the participants stood still in an upright position. The subjects only lifted the box from floor to table or from table to table, while the test leader moved the box back to the starting position, i.e., the subjects only lifted the box in the lifting phase. The table height and reaching distance were the same for all subjects and were not relative to each participant. The reason for this was that we wanted to simulate a lifting situation similar to a working site where the workers rarely have the possibility of adapting the lifting tasks to their individual height. The test leader visually inspected every lift, and the trial was excluded if it was performed in an uncontrolled manner regarding jerky movements or high lifting pace. We selected concentric phases only as higher sEMG is reported during muscle shortening contractions compared with eccentric phases [18, 19]. Two tables were placed in a 90° angle, and the subjects were always moving the load from left to right. The lifting conditions are illustrated in Fig. 1 and were performed in the following manner:

1. lifting 3, 15 and 30 kg with forearm length reaching distance (short reaching distance), i.e., the length from body center of mass to the center of mass of the box corresponding forearm length, while moving the feet and without rotation of the trunk (Fig. 1a-d).
2. lifting 3 and 15 kg with a ¾ arm length reaching distance (long reaching distance) while moving the feet and without rotation of the trunk (Fig. 1e-h).
3. lifting 3 and 15 kg with a forearm length reaching distance with trunk rotation and without moving the feet (Fig. 1i-l).

The weights were determined on the basis of the recommendations made by The Danish Working Agency [20]. Familiarization was achieved by performing a few test trials with each load and for each condition. Then, three trials were recorded for each load. Each condition had a minimum of 1 min of rest between each lifting trial. The lifts were performed in a randomized counter-balanced order. The randomization was blinded to the experimenter, and each subject drew a sealed, unmarked envelope with the order of the lifts to be performed. Once the envelope had been opened, the order was noted by the test leader and could not be changed. Thus, when the envelope had been opened, the experimenter was not blinded. The same order was used for each subject during the two test sessions.

### Surface electromyography recordings and analysis

**Fig. 2:**
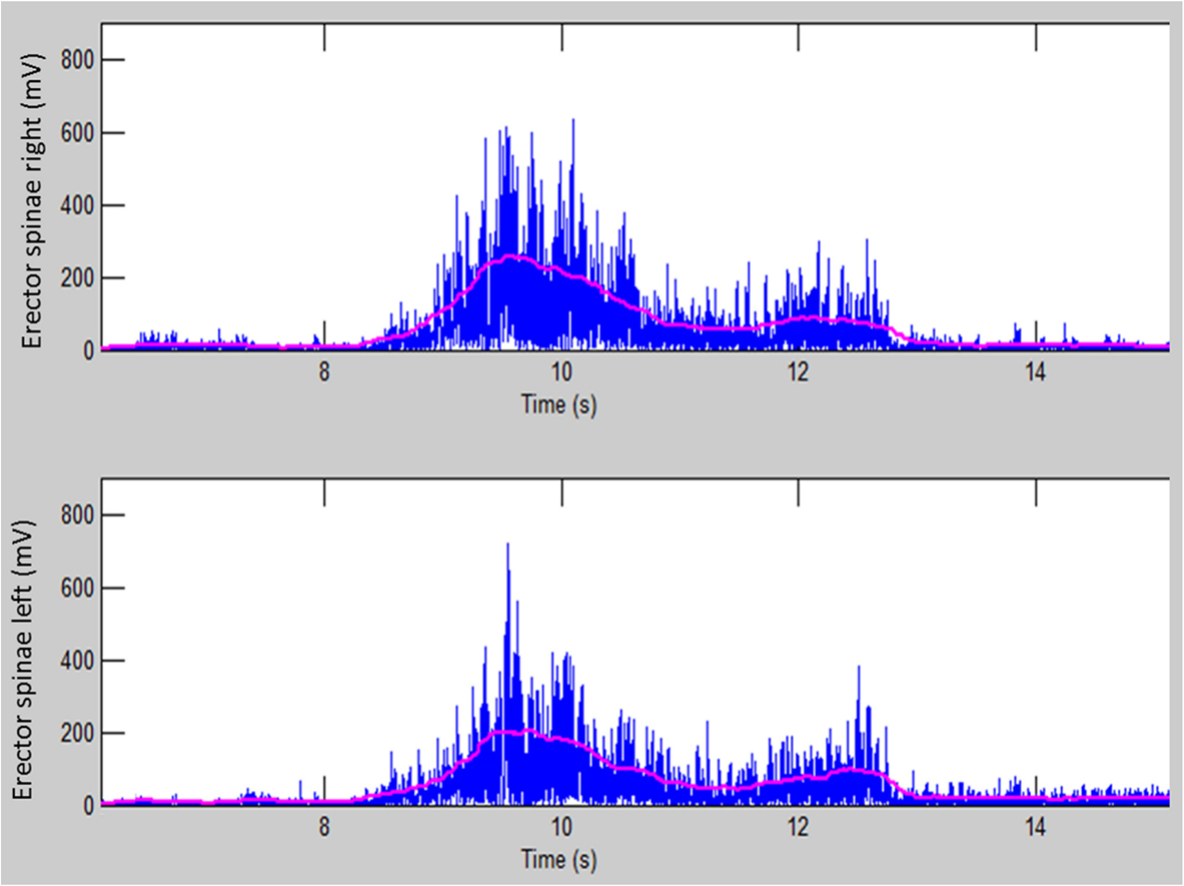
Example of the sEMG (mV) from erector spinae descendens (left and right) during standardized lift from floor to table in the forearm condition with a load of 30 kg. The *blue* line shows rectification of the sEMG and the pink line shows the filtered sEMG

The placement of surface electrodes and the recording of the sEMG followed the SENIAM guidelines (http://www.seniam.org/) and the standard for reporting sEMG (http://www.isek.org/emg-standards/). All electrodes were placed by the same experienced test leader on both test days. The test leader had experience with the procedure of placing electrodes and was careful to palpate the anatomical landmarks to ensure the correct placement according to the SENIAM guidelines (http://www.seniam.org). Bipolar sEMG electrodes (Blue Sensor N-00-S/25, Ambu A/S, Ballerup, Denmark) (skin contact size 30 * 20 mm) were placed longitudinally to the muscle fibers with an inter-electrode distance of 2 cm [21] over the left and right trapezius on the shoulder and the left and right erector spinae on the low back [11]. The electrodes for the trapezius muscle were placed bilaterally, ~20% lateral to the midpoint between the acromion and the C7 vertebra of the descending part of the trapezius muscles, and two finger widths (corresponding to ~2.5 cm) lateral from the proc. spine of L1 for the erector spinae muscles. A reference electrode was placed above the C7 vertebra. Before mounting of the sEMG electrodes, the skin of the subject was shaved and prepared using scrubbing gel (Acqua gel, Meditec, Parma, Italy) to lower the skin-electrode impedance. The cables were fixed with tape (Fixomull stretch) to ensure durability and to minimize the potential inconvenience for the subjects. The bipolar sEMG signals were amplified 19.5 times and sampled at 1024 Hz using a 24-bit portable data-logger (Input impedance >10^12^ Ω, CMRR: 100 dB, Nexus10, Mind Media, Netherlands). sEMG recordings were analyzed in Matlab (MathWorks, Natick, MA, USA) using a custom-made program. The sEMGs were digitally filtered (using a [10-400] Hz, 2nd order zero-phase Butterworth band-pass filter and a Notch filter with a width of 1 Hz at a frequency of 50 Hz). Figure 2 shows an example of the sEMG during a standardized lift. The root mean square (RMS) values were calculated over epochs of 500 ms with 20% overlap between successive epochs for both MVCs and lifting tasks. For MVC recordings, the maximal amplitude, denoted as RMS_max_, was obtained for each MVC repetition and then the highest RMS value of the three repetitions was extracted and used for reliability and normalization purposes [22]. Further, the maximal amplitude was extracted from each standardized lifting task. Then, the absolute and normalized RMS (absRMS and normRMS) data were computed and saved for statistical analyses.

### Statistics

The relative and absolute reliability of absRMS and normRMS across the lifts were computed using ICC_3,k_, SEM and MDC. The ICC_3,k_ was calculated according to the method of Shrout-Fleiss [23]. The ICC_3,k_ values were interpreted using the categories proposed previously in which an ICC between 0.00-0.20 is considered poor, 0.21-0.40 is fair, 0.41-0.60 is moderate, 0.61-0.80 is substantial, and 0.81-1.00 is almost perfect [24]. The SEM was calculated as standard deviation (SD) of the test scores multiplied by the square root of 1 – ICC [15].

Equation 1:

$$ {ICC}_{3.K}=\frac{MS_S-{MS}_E\ }{MS_s} $$

Where MS_S_ = subjects mean square, MS_E_ = Error mean square.

Equation 2:

$$ SEM= SD\sqrt{1}- ICC $$

Equation 3:

$$ SEM\%={\frac{SEM}{Mean}}^{\ast }100 $$

The MDC was calculated as SEM times 1.96 times the square root of 2 [25].

Equation 4:

$$ MDC={SEM}^{\ast }1.96\ \sqrt{2} $$

Equation 5:

$$ MDC\%={\frac{MDC}{Mean}}^{\ast }100 $$

As a secondary analysis, a student’s t-test and Pearson’s correlations coefficient (Microsoft, Excel) were used to comparing RMS_max_ values from MVCs from day one and day two.

## Results

The absRMS and normRMS values are presented in Tables 3 and 4. No significant difference was found between absRMS or normRMS values recorded on day 1 and day 2. The inter-day ICCs of the absRMS for the erector spinae muscles ranged between 0.51 to 0.92 and 0.63 to 0.92 for the normRMS during lifts from table to table. For lifts from floor to table, the absRMS ranged from 0.67 to 0.93, and the normRMS ranged from 0.21 to 0.76 (Table 3). Similar values were obtained for the left and right erector spinae muscles. For the trapezius muscles, the ICCs of the absRMS ranged between 0.08 to 0.89 and 0.11 and 0.83 for normRMS for lifts performed from table to table. For lifts from floor to table, the absRMS ranged between 0.35 to 0.88, and the normRMS ranged from 0.28 to 0.76 (Table 4). In general, ICC values were higher (i) for absRMS compared with normRMS and (ii) for the right trapezius compared with the left trapezius. The overall ICC for absolute and normalized erector spinae sEMG was 0.81 (95% CI 0.75-0.86) and 0.83 (95% CI 0.79-0.87), respectively, for the table to table condition, and 0.85 (95% CI 0.82-0.89) and 0.49 (95% CI 0.41-0.57), respectively, for the floor to table condition. The overall ICC for absolute and normalized trapezius sEMG was 0.58 (95% CI 0.44-0.72) and 0.46 (95% CI 0.34-0.57), respectively, for the table to table condition, and 0.67 (95% CI 0.59-0.76) and 0.51 (95% CI 0.43-0.58), respectively, for the floor to table condition. Of all the lifting situations, three lifting situations were considered poor, three were fair, eight were moderate, 12 were substantial, and 30 were almost perfect for the absRMS. For normRMS, three lifting situations ICCs were considered poor, 12 were fair, 14 were moderate, 17 were substantial, and ten were almost perfect. Thus, a total of 50 out of 56, i.e., 89%, and 41 out of 56, i.e., 73%, of the lifting situations, were in the range from moderate to almost perfect for absRMS and normRMS, respectively. The SEM, SEM%, MDC and MDC% are presented in Tables 5 and 6. In general, these figures were lower for a lift from table to table than from floor to table and similarly for absRMS and normRMS.

**Table 3:** Mean ± SD absolute and normalized root mean square values (absRMS (mV) and normRMS (% of the highest RMS values of the three maximum voluntary contractions)) of the left and right erector spinae surface electromyograms during standardized box lifting at day 1 and day 2, intraclass correlation coefficient (ICC3.K) values and between-day normRMS difference for left and right erector spinae

	Erector Spinae							
	Absolute sEMG				Normalised sEMG			
Lifting condition	Day 1 (mV)	Day 2 (mV)	ICC3.K	Difference (mV)	Day 1 (mV)	Day 2 (mV)	ICC3.K	Difference (mV)
Table to Table 3 kg								
Short reaching distance								
Left	41.2 ± 10.0	41.1 ± 12.1	0.79	−0.1 ± 9.2	14.1 ± 4.5	14.3 ± 5.0	0.92	0.2 ± 2.8
Right	43.7 ± 17.6	45.3 ± 15.6	0.64	1.6 ± 17.1	15.3 ± 6.0	16.3 ± 9.1	0.77	1.0 ± 6.7
Long reaching distance								
Left	52.0 ± 14.9	50.3 ± 16.1	0.90	−1.7 ± 9.1	17.4 ± 5.5	17.5 ± 6.8	0.91	0.1 ± 3.5
Right	52.0 ± 20.4	52.0 ± 16.1	0.82	−0.0 ± 14.5	18.0 ± 6.1	17.8 ± 5.6	0.77	−0.2 ± 5.1
Trunk rotation								
Left	57.4 ± 22.0	57.0 ± 18.2	0.88	−0.4 ± 13.4	18.8 ± 6.4	19.4 ± 5.9	0.86	0.6 ± 4.3
Right	37.2 ± 19.9	31.7 ± 9.6	0.71	−5.5 ± 14.8	12.6 ± 5.6	10.8 ± 3.0	0.80	−1.8 ± 3.7
Table to Table 15 kg								
Short reaching distance								
Left	78.5 ± 25.7	72.9 ± 20.3	0.80	−5.6 ± 19.0	26.3 ± 9.3	25.0 ± 7.4	0.82	−1.3 ± 6.5
Right	77.3 ± 30.1	77.8 ± 19.9	0.88	0.5 ± 17.0	26.9 ± 9.3	26.8 ± 8.3	0.88	−0.1 ± 5.7
Long reaching distance								
Left	104.6 ± 34.4	96.7 ± 29.1	0.92	−7.9 ± 17.8	34.5 ± 11.5	32.6 ± 9.2	0.85	−1.9 ± 7.6
Right	105.1 ± 42.8	99.4 ± 27.9	0.83	−5.7 ± 27.6	35.8 ± 10.5	33.8 ± 9.7	0.88	−2.0 ± 6.6
Trunk rotation								
Left	94.3 ± 33.8	97.5 ± 35.1	0.86	3.2 ± 24.2	30.9 ± 10.0	33.0 ± 10.0	0.86	2.1 ± 6.9
Right	71.2 ± 50.1	66.9 ± 19.7	0.51	−4.3 ± 43.6	23.7 ± 11.9	23.2 ± 9.4	0.63	−0.5 ± 11.1
Table to Table 30 kg								
Short reaching distance								
Left	123.5 ± 40.9	112.4 ± 33.6	0.88	−11.1 ± 24.7	41.3 ± 15.2	38.0 ± 10.4	0.79	−3.3 ± 10.9
Right	124.2 ± 55.2	117.2 ± 35.4	0.88	−7.0 ± 30.0	42.5 ± 14.2	40.1 ± 13.4	0.90	−2.4 ± 8.5
Floor to Table 3 kg								
Short reaching distance								
Left	125.5 ± 57.4	117.9 ± 44.8	0.91	−7.6 ± 29.4	38.8 ± 7.1	38.8 ± 9.8	0.76	0.0 ± 7.5
Right	135.1 ± 62.2	117.2 ± 42.0	0.88	−17.9 ± 34.3	45.0 ± 12.2	39.0 ± 9.6	0.75	−6.0 ± 9.8
Long reaching distance								
Left	135.2 ± 60.9	117.9 ± 37.2	0.83	−17.3 ± 38.9	42.0 ± 8.9	39.3 ± 10.0	0.34	−2.7 ± 11.9
Right	131.5 ± 57.3	120.2 ± 38.1	0.72	−11.3 ± 45.7	44.0 ± 11.4	40.3 ± 9.7	0.38	−3.7 ± 13.1
Trunk rotation								
Left	122.8 ± 54.5	116.0 ± 35.6	0.89	−6.8 ± 28.6	38.2 ± 8.3	38.5 ± 8.1	0.65	0.3 ± 8.4
Right	122.3 ± 54.1	120.6 ± 31.7	0.67	−1.7 ± 44.2	41.1 ± 10.8	41.2 ± 11.3	0.50	0.1 ± 12.8
Floor to Table 15 kg								
Short reaching distance								
Left	176.4 ± 69.9	172.4 ± 60.5	0.93	−4.0 ± 34.2	55.4 ± 10.8	57.1 ± 14.5	0.59	1.7 ± 13.8
Right	181.2 ± 73.3	173.6 ± 58.8	0.88	−7.6 ± 44.3	60.3 ± 11.5	57.8 ± 13.5	0.48	−2.5 ± 14.6
Long reaching distance								
Left	195.0 ± 77.4	183.1 ± 56.3	0.87	−11.9 ± 45.2	61.4 ± 13.1	61.1 ± 15.9	0.21	−0.3 ± 19.3
Right	197.1 ± 84.8	187.2 ± 66.6	0.85	−9.9 ± 55.5	65.5 ± 14.3	62.2 ± 15.4	0.33	−3.3 ± 18.8
Trunk rotation								
Left	172.0 ± 55.8	165.3 ± 46.1	0.91	−6.7 ± 28.9	55.1 ± 10.1	55.3 ± 11.6	0.51	0.2 ± 12.4
Right	179.3 ± 70.7	169.8 ± 45.9	0.88	−9.5 ± 39.6	59.8 ± 12.4	57.5 ± 13.3	0.59	−2.3 ± 13.8
Floor to Table 30 kg								
Short reaching distance								
Left	228.2 ± 84.7	209.3 ± 69.1	0.86	−18.9 ± 53.4	72.1 ± 13.8	70.5 ± 21.2	0.30	−1.6 ± 22.9
Right	235.1 ± 95.0	214.1 ± 74.1	0.85	−21.0 ± 62.3	77.7 ± 13.9	71.2 ± 17.4	0.46	−6.5 ± 18.6

Difference = difference in absRMS (mV) and normRMS (%) between day 1 and day 2. Forearm length = short distance, ¾ arm distance = long distance and trunk rotation = short distance with trunk rotation

**Table 4:** Mean ± SD absolute and normalized root mean square values (absRMS (mV) and normRMS (% of the highest RMS values of the three maximum voluntary contractions)) of the left and right trapezius descendens surface electromyograms during standardized box lifting at day 1 and day 2, intraclass correlation coefficient (ICC3.K) values and between-day normRMS difference for left and right erector spinae

	Trapezius							
	Absolute sEMG				Normalised sEMG			
Lifting condition	Day 1 (mV)	Day 2 (mV)	ICC3.K	Difference (mV)	Day 1 (mV)	Day 2 (mV)	ICC3.K	Difference (mV)
Table to Table 3 kg								
Short reaching distance								
Left	66.6 ± 36.0	59.7 ± 37.9	0.19	−6.9 ± 49.4	10.0 ± 6.5	11.0 ± 7.0	0.18	1.0 ± 9.1
Right	80.2 ± 51.4	69.9 ± 36.5	0.87	−10.3 ± 30.0	11.2 ± 7.1	10.9 ± 6.2	0.64	−0.3 ± 6.8
Long reaching distance								
Left	90.7 ± 57.3	70.8 ± 48.2	0.31	−19.9 ± 67.6	13.3 ± 9.7	12.4 ± 6.8	0.23	−0.9 ± 11.1
Right	90.0 ± 69.3	84.9 ± 40.3	0.78	−5.1 ± 47.7	12.8 ± 9.2	13.5 ± 8.0	0.62	0.7 ± 9.1
Trunk rotation								
Left	71.9 ± 51.5	53.0 ± 34.8	0.08	−18.9 ± 60.7	10.8 ± 8.7	9.6 ± 6.4	0.12	−1.2 ± 10.5
Right	93.8 ± 67.7	76.9 ± 48.0	0.84	−16.9 ± 43.1	13.0 ± 9.2	12.0 ± 8.1	0.64	−1.0 ± 8.9
Table to Table 15 kg								
Short reaching distance								
Left	148.7 ± 89.1	126.0 ± 78.0	0.14	−22.7 ± 113.8	21.7 ± 14.5	21.5 ± 9.5	0.40	−0.2 ± 15.0
Right	169.0 ± 91.9	156.3 ± 84.7	0.77	−12.7 ± 76.7	23.2 ± 11.9	24.4 ± 17.7	0.62	1.1 ± 15.8
Long reaching distance								
Left	231.4 ± 156.3	187.0 ± 111.3	0.57	−44.4 ± 148.5	32.4 ± 22.9	32.4 ± 15.3	0.36	0.0 ± 24.4
Right	267.0 ± 175.8	223.1 ± 121.3	0.83	−43.9 ± 114.9	36.1 ± 20.9	36.5 ± 28.5	0.76	0.4 ± 21.8
Trunk rotation								
Left	153.6 ± 120.3	124.1 ± 72.1	0.52	−29.5 ± 112.7	21.4 ± 17.2	20.6 ± 8.7	0.11	−0.8 ± 18.7
Right	216.6 ± 157.3	182.4 ± 125.6	0.89	−34.2 ± 88.3	32.0 ± 26.6	30.0 ± 28.8	0.57	−2.0 ± 30.5
Table to Table 30 kg								
Short reaching distance								
Left	308.0 ± 223.7	236.0 ± 128.4	0.47	−72.0 ± 214.2	43.2 ± 32.1	38.4 ± 13.3	0.30	−4.8 ± 31.5
Right	344.7 ± 250.3	281.8 ± 160.0	0.84	−62.9 ± 157.9	46.9 ± 29.6	43.9 ± 35.3	0.83	−3.0 ± 24.6
Floor to Table 3 kg								
Short reaching distance								
Left	78.9 ± 45.6	60.7 ± 41.0	0.36	−18.2 ± 54.2	11.7 ± 7.6	11.4 ± 7.9	0.37	−0.3 ± 9.7
Right	81.0 ± 69.4	75.5 ± 36.7	0.80	−5.5 ± 45.3	10.9 ± 8.2	11.7 ± 6.5	0.76	0.8 ± 6.5
Long reaching distance								
Left	91.3 ± 60.3	75.4 ± 56.6	0.60	−15.9 ± 62.5	13.5 ± 10.5	13.6 ± 10.2	0.49	0.1 ± 12.1
Right	86.9 ± 61.7	81.9 ± 46.9	0.73	−5.0 ± 50.6	12.0 ± 8.1	13.5 ± 9.7	0.66	1.5 ± 9.0
Trunk rotation								
Left	70.1 ± 41.2	61.7 ± 41.7	0.35	−8.4 ± 52.2	10.0 ± 5.7	11.0 ± 7.3	0.31	1.0 ± 8.4
Right	92.6 ± 70.8	85.2 ± 49.4	0.80	−7.4 ± 49.8	12.2 ± 8.2	12.9 ± 8.5	0.74	0.7 ± 7.6
Floor to Table 15 kg								
Short reaching distance								
Left	160.0 ± 96.3	156.8 ± 80.1	0.53	−3.2 ± 100.1	22.8 ± 14.2	28.7 ± 16.6	0.28	5.9 ± 20.0
Right	178.3 ± 114.1	174.9 ± 85.3	0.86	−3.4 ± 89.5	26.2 ± 19.3	27.4 ± 18.7	0.58	1.2 ± 20.7
Long reaching distance								
Left	216.2 ± 128.4	181.2 ± 92.0	0.67	−35.0 ± 111.6	30.1 ± 17.7	32.3 ± 17.2	0.33	2.2 ± 22.1
Right	232.3 ± 134.7	228.3 ± 136.0	0.88	−4.0 ± 69.6	35.3 ± 28.8	36.9 ± 31.3	0.45	1.6 ± 35.8
Trunk rotation								
Left	177.6 ± 101.4	155.7 ± 77.3	0.59	−21.9 ± 97.5	25.0 ± 14.3	27.9 ± 15.1	0.42	2.9 ± 17.8
Right	241.5 ± 156.2	193.0 ± 102.1	0.84	−48.5 ± 99.2	35.5 ± 26.0	31.5 ± 23.5	0.67	−4.0 ± 24.8
Floor to Table 30 kg								
Short reaching distance								
Left	337.5 ± 203.4	304.2 ± 176.7	0.55	−33.3 ± 212.1	45.3 ± 23.9	53.8 ± 30.9	0.50	8.5 ± 31.8
Right	336.1 ± 171.7	303.5 ± 171.8	0.83	−32.6 ± 131.1	47.9 ± 28.0	49.3 ± 41.0	0.53	1.4 ± 39.7

Difference = difference in absRMS (mV) and normRMS (%) between day 1 and day 2. Forearm length = short distance, ¾ arm distance = long distance and trunk rotation = short distance with trunk rotation

**Table 5:** Standard error of measurement (SEM (mV)), standard error of measurement in percent (SEM% (%)), minimal detectable change (MDC (mV)) and minimal detectable change in percent (MDC% (%)) of the absolute and normalized root mean square values of the left and right erector spinae surface electromyography during standardized box lifting

	Erector Spinae							
	Absolute sEMG				Normalised sEMG			
Lifting condition	SEM (mV)	SEM % (%)	MDC (mV)	MDC % (%)	SEM (mV)	SEM % (%)	MDC (mV)	MDC % (%)
Table to Table 3 kg								
Short reaching distance								
Left	5.0	12.2	13.9	33.9	1.5	10.2	4.0	28.3
Right	10.0	22.4	27.6	62.0	3.7	23.6	10.3	65.3
Long reaching distance								
Left	4.8	9.4	13.3	26.0	1.8	10.5	5.1	29.2
Right	7.9	15.2	21.9	42.0	2.9	15.9	7.9	44.1
Trunk rotation								
Left	7.1	12.4	19.7	34.4	2.3	11.9	6.3	33.0
Right	8.6	24.8	23.7	68.8	2.1	17.5	5.7	48.5
Table to Table 15 kg								
Short reaching distance								
Left	10.5	13.8	29.0	38.4	3.6	13.9	9.9	38.4
Right	9.0	11.6	24.9	32.1	3.0	11.2	8.4	31.1
Long reaching distance								
Left	9.4	9.3	25.9	25.8	4.1	12.1	11.3	33.6
Right	15.0	14.6	41.4	40.5	3.5	10.2	9.8	28.1
Trunk rotation								
Left	12.9	13.5	35.8	37.4	3.7	11.6	10.3	32.3
Right	26.7	38.6	73.9	107.0	6.5	27.7	18.0	76.8
Table to Table 30 kg								
Short reaching distance								
Left	13.2	11.2	36.6	31.0	6.0	15.2	16.7	42.0
Right	15.9	13.2	44.1	36.5	4.5	10.9	12.4	30.1
Floor to Table 3 kg								
Short reaching distance								
Left	15.4	12.7	42.7	35.1	4.2	10.8	11.6	29.9
Right	18.4	14.6	51.0	40.4	5.7	13.5	15.7	37.3
Long reaching distance								
Left	21.4	16.9	59.4	46.9	7.8	19.1	21.5	52.9
Right	26.1	20.7	72.1	57.4	8.4	20.0	23.4	55.4
Trunk rotation								
Left	15.1	12.6	41.8	35.0	4.9	12.7	13.5	35.3
Right	25.5	21.0	70.8	58.3	7.8	19.0	21.7	52.7
Floor to Table 15 kg								
Short reaching distance								
Left	17.7	10.2	49.2	28.2	8.2	14.6	22.7	40.3
Right	23.5	13.3	65.2	36.8	9.1	15.3	25.1	42.5
Long reaching distance								
Left	24.1	12.7	66.8	35.3	12.9	21.0	35.7	58.3
Right	29.9	15.9	82.8	43.1	12.3	19.2	34.0	53.2
Trunk rotation								
Left	15.1	8.9	41.8	24.8	7.6	13.7	21.0	38.1
Right	21.1	12.1	58.4	33.5	8.2	14.0	22.8	38.9
Floor to Table 30 kg								
Short reaching distance								
Left	28.7	13.1	79.5	36.3	15.0	21.0	41.5	58.1
Right	33.8	15.0	93.6	41.7	11.8	15.9	32.7	44.0

Forearm length = short distance, ¾ arm distance = long distance and trunk rotation = short distance with trunk rotation

**Table 6:** Standard error of measurement (SEM (mV)), standard error of measurement in percent (SEM% (%)), minimal detectable change (MDC (mV)) and minimal detectable change in percent (MDC% (%)) of the absolute and normalized root mean square values of the left and right trapezius descendens surface electromyography during standardized box lifting

	Trapezius							
	Absolute sEMG				Normalised sEMG			
Lifting condition	SEM (mV)	SEM % (%)	MDC (mV)	MDC % (%)	SEM (mV)	SEM % (%)	MDC (mV)	MDC % (%)
Table to Table 3 kg								
Short reaching distance								
Left	33.4	52.9	92.5	146.6	6.1	58.4	17.0	162.0
Right	16.1	21.4	44.5	59.3	4.0	36.1	11.4	100.0
Long reaching distance								
Left	44.7	55.3	123.8	153.3	7.4	57.2	20.4	158.5
Right	26.3	30.1	73.0	83.5	5.3	40.6	14.8	112.5
Trunk rotation								
Left	43.0	68.9	119.2	190.9	7.2	70.4	19.9	195.2
Right	23.4	27.4	64.9	76.1	5.2	41.4	14.3	114.7
Table to Table 15 kg								
Short reaching distance								
Left	78.3	57.0	216.9	157.9	9.5	44.2	26.4	122.4
Right	42.7	26.2	118.3	72.7	9.3	39.0	25.7	108.1
Long reaching distance								
Left	89.9	43.0	249.2	119.1	15.6	48.2	43.1	133.5
Right	62.8	25.6	174.0	71.0	12.1	33.5	33.7	92.8
Trunk rotation								
Left	69.3	49.9	192.0	138.3	12.9	61.3	35.6	169.9
Right	46.8	23.5	129.7	65.0	18.3	59.0	50.6	163.4
Table to Table 30 kg								
Short reaching distance								
Left	135.0	49.6	374.1	137.5	20.6	50.5	57.1	139.9
Right	86.2	27.5	238.9	76.3	13.3	29.3	36.8	81.1
Floor to Table 3 kg								
Short reaching distance								
Left	35.5	50.9	98.4	141.0	6.2	53.5	17.1	148.2
Right	24.8	31.7	68.8	87.9	3.6	32.2	10.1	89.1
Long reaching distance								
Left	37.4	44.8	103.5	124.2	7.4	54.7	20.6	151.7
Right	28.5	33.8	79.0	93.7	5.2	41.1	14.5	113.8
Trunk rotation								
Left	33.7	51.2	93.5	141.9	5.5	52.0	15.1	144.3
Right	27.3	30.7	75.7	85.1	4.3	33.8	11.8	93.7
Floor to Table 15 kg								
Short reaching distance								
Left	60.7	38.3	168.2	106.2	13.3	51.8	36.9	143.5
Right	37.1	21.0	102.8	58.2	12.3	45.9	34.1	127.3
Long reaching distance								
Left	65.2	32.8	180.7	91.0	14.3	45.9	39.7	127.3
Right	47.4	20.6	131.4	57.1	22.2	61.6	61.6	170.7
Trunk rotation								
Left	58.4	35.0	161.8	97.1	11.3	42.6	31.2	118.0
Right	54.4	25.1	150.8	69.4	14.4	43.0	39.9	119.2
Floor to Table 30 kg								
Short reaching distance								
Left	128.2	40.0	355.3	110.8	19.7	39.7	54.5	110.0
Right	71.2	22.3	197.5	61.8	24.0	49.5	66.6	137.1

Forearm length = short distance, ¾ arm distance = long distance and trunk rotation = short distance with trunk rotation

**Fig. 3:**
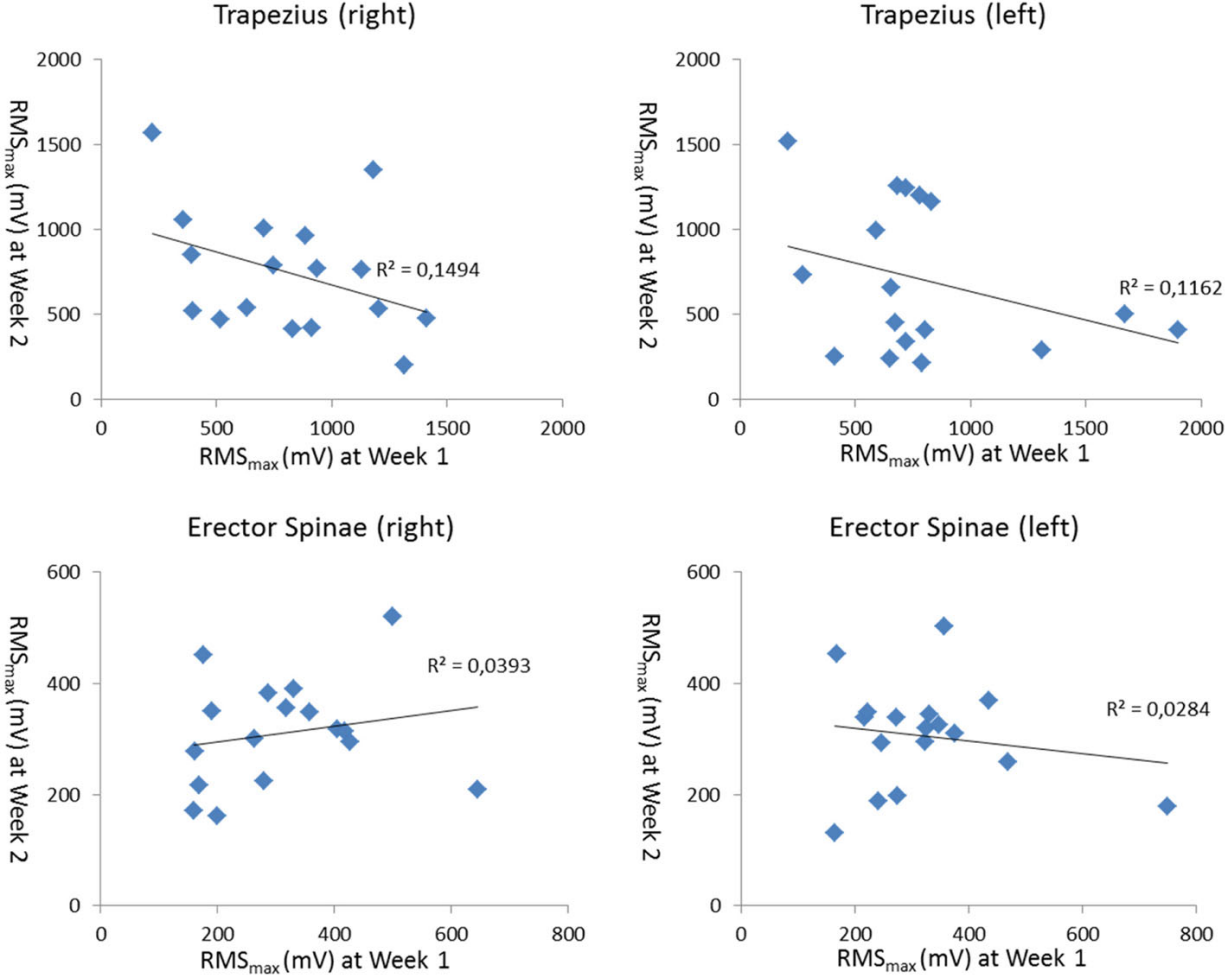
Maximum absolute RMS values (RMSmax (mV)) extracted from maximal voluntary contraction of the right and left trapezius and right and left erector spinae extracted from maximal voluntary contraction at week one (x-axis) and week two (y-axis)

For the MVCs, the mean RMS_max_ values for the right and left trapezius were 812 mV (± 360 mV), 747 mV (± 360 mV) and 804 mV (± 442 mV), 699 (± 437 mV) on day one and two, respectively. For the right and left erector spinae the values on day one and two were 311 mV (± 136 mV), 311 (± 97 mV), 324 mV (± 139 mV) and 305 (± 96 mV), respectively. No significant difference was found between day one and day two (*P* > 0.66). The Pearson’s r values were −0.39, −0.34, 0.20, and −0.17 for the right trapezius, left trapezius, right erector spinae and left erector spinae, respectively (Fig. 3).

## Discussion

This reliability study showed that absolute and normalized maximum sEMG amplitudes obtained during box lifts have a moderate to substantial inter-day reliability for most lifts, but were more reliable for box lifts from table to table than from floor to table both for trapezius and erector spinae muscles. In addition, absRMS values were found more reliable than normRMS regarding relative reliability and similarly with respect to absolute reliability. In general, absRMS and normRMS for the erector spinae muscles were more reliable than for the trapezius muscles.

### Relative reliability of absolute and normalized sEMG recordings during standardized lifts

Reliability should be expressed regarding relative and absolute reliability (Weir, 2005). In an sEMG context, the relative reliability can express the degree at which participants maintain their ranking of the level of muscle activation during repeated measurements. The absolute reliability corresponds to the degree to which repeated measurements vary for participants [26]. Consequently, the relative reliability is affected by the ratio of the variability between participants and the total variability [27] meaning that high ICC values can be found in a heterogeneous group of participants [15]. As opposed to this, the absolute reliability estimated by calculating, e.g., SEM and MDC are not affected by the total variability as it is related to the difference within each participant across repeated measurements [22]. In this study, we extracted the maximal amplitude of the sEMG from the erector spinae and trapezius muscle to assess the reliability of the maximum muscular load during standardized lifts. Such information is of interest for intervention studies aiming at reducing events with excessive physical load [14]. Of note, in ergonomics, the 10th, 50th and 90th percentiles of the sEMG RMS distributions are often used for characterizing sEMG [28]. Future studies could address the reliability of the 10th, 50th and 90th percentiles. The relative reliability of the sEMG maximum amplitudes was influenced by the normalization procedure. We found higher ICCs for absolute compared with normalized amplitudes in line with previous studies [22, 29–32]. In a systematic review, it was concluded that normalization of sEMG with respect to values measured during MVCs should be preferred in healthy individuals [33]. However, this choice can be questioned when assessing dynamic contractions like standardized lifts. As such, submaximal contractions are also suggested as suitable when aiming at reducing within and between subjects’ variations [34–36]. Another important aspect lies in the fact that normalization of the sEMG also increases the magnitude of variance [37, 38]. In the present study, the latter is substantiated by the low correlation between the RMS_max_ extracted from the MVCs (Fig. 3). The fact that the MVC tests were performed with resistance from the test leader may have caused extra variance. Furthermore, the fact that the MVCs were recorded during an isometric condition while the maximum RMS during standardized lifts occurred during concentric muscle action can also partly explain the difference in ICC due to the volume conductor issue [39]. All in all, the higher ICCs found for absolute compared with normalized RMS mostly underline the larger heterogeneity of the RMS values extracted from the MVCs.

In the present study, the different lifting conditions influenced the reliability of the sEMG measurements. In general, the highest relative reliability was found for lifting from table to table, e.g., for the erector spinae muscles the ICCs were generally above 0.61 (except one) corresponding to at least moderate reliability [24]. However, the reliability of the lifting from floor to table varied in a wide range depending on the muscle and load in question with ICCs ranging from 0.08 to 0.93, i.e., from fair to substantial [24]. The lifting condition from floor to table was more difficult to standardize and reproduce because the subjects had to perform the lifting task over several body segments by flexing and extending the ankles, hips and knees, which at the same time leads to higher muscle load as documented by the higher normRMS values (Tables 3 and 4). It could be speculated that the subjects chose a motor control solution with an increased viable coordination plan between the joints/muscles. The stretch of the hamstring and lower back during a lift from floor to table may also make the lift more uncomfortable and difficult to reproduce. Further, the pull of the skin associated with bending over and lifting a load from the floor may have affected the sEMG electrodes on the back and caused a noisy sEMG signal due to skin electrode artifacts. Furthermore, the volume conductor effect in which the distance from the motor units to the sEMG electrode or the amount of motor units from which the effect was measured can change during dynamic sEMG recordings due to skin movement during dynamic contraction [39, 40] and may have had influenced the results. Altogether, these factors may explain the lower reliability of lifting from floor to table compared with table to table. Further, the addition of kinematics measurements to sEMG may be necessary to obtain reliable estimates of the loads lifted from the floor to table.

In general, the ICCs for the right trapezius muscle in this study were in line with a previous study measuring the sEMG of the trapezius during MVCs in several positions [22]. For the left trapezius, the ICCs were lower than for the right trapezius. In the present study, 16 participants were right-handed, and one was left-handed, and it is possible that it is easier to reproduce a lifting task performed with the dominant trapezius muscle than with the non-dominant. As mentioned above, Andersen et al. (2014) also reported higher ICCs for absolute sEMG values from the trapezius compared with normalized values during isometric flexion, abduction, and internal and external rotation of the shoulder [22]. In the present study, we reported both absolute and normalized RMS values and found differences in the relative reliability of absolute and normalized sEMG amplitudes in healthy participants performing standardized lifting tasks. As mentioned by Januario et al. (2016), future sEMG studies need to further assess normalization aspects [41].

The loads lifted, i.e., 3, 15 and 30 kg, did not markedly influence the reliability of the sEMG measurements (Tables 3 and 4). This is important in relation to real-life working conditions in which both low and high workloads occur during the work day. As expected, a clear lifting load sEMG relationship was found, i.e., heavier loads resulted in higher sEMG amplitudes (Tables 3 and 4).

### Absolute reliability of absolute and normalized sEMG recordings during standardized lifts

The absolute reliability of the absRMS and normRMS depicted by SEM% values ranged from 8.94 to 38.61% and from 10.15 to 27.69%, respectively, for the erector spinae muscles and from 20.58 to 68.89% and from 29.26 to 70.44% for the trapezius muscles (Table 4). The SEM was higher for the trapezius muscles than for the erector spinae muscles and higher when performing lifts from floor to table than when performing lifts from table to table. For intervention purposes, this suggests that the true normRMS was below or above measured normRMS with between 1.45 and 14.95% for the erector spinae muscles and between 3.63 and 24.03% for the trapezius muscles. Furthermore, this suggests that a clinical change will not be masked by the standard error of measurement if the normRMS from an intervention changes by more than 14.95% for the erector spinae muscles and 24.03% for the trapezius muscles. Such information is extremely important when assessing the effects of, e.g., ergonomics interventions [42]. In the present study, the absolute reliability of the absolute and normalized sEMG amplitudes was similar in line with [22]. The number of published studies assessing absolute reliability during lifting tasks is very limited in the literature, which makes comparisons difficult. We found one study reporting SEMs of 9.9% and 20.3% in the erector spinae during maximal flexion of the back and maximal rotation of the trunk, respectively [31]. For the trapezius, Michener et al. (2016) reported SEMs ranging from 5.5 to 24.9% during arm elevation and lowering in the scapular plane [30]. All in all, the SEMs reported during box lifting tasks are within the ranges reported by Schinkel-Ivy et al. (2015) [31] and Michener et al. (2016) [30].

### Strengths and limitations

The sEMG for the present study was performed with the purpose of finding the peak sEMG during the box lifts. Thus, it would have been helpful to precisely divide the lifting movement in their concentric and eccentric phase. We did opt for that for two reasons: 1) The test leader always lowered the box to the starting position and therefore there was no eccentric phase with the external load during the lifts. 2) We aimed at applying the approach in a participative ergonomic intervention. In this randomized controlled trial, we wished to detect the working situations with the highest physical loading regarding high muscular activity based on an entire working day of recordings [14]. Furthermore, a previous study has shown that the peak sEMG appears in the concentric phase for the erector spinae during repetitive lifting [19].

The recording and processing of the sEMG followed the SENIAM guidelines and ISEK recommendations, and all measurements were carried out by the same experienced test leader. However, we cannot reject some variation with respect to placement of electrodes. This issue is inevitable and common in longitudinal studies. Furthermore, we only recorded sEMG from erector spinae and trapezius. Even though the experimenter checked the quality of all lifts, visually detecting differences in movements and lifting velocity between the different lifts, days and subjects can be difficult. This was especially the case when the lifts were performed with light loads, which are inherently more prone to faster movements. In this study, the participants performed test trials of the lifts before recording the trials, but an entire familiarization session before the actual test day might have increased the reliability. We selected healthy male participants to ensure a homogenous group as workers often report pain [43, 44] known to affect the ability to perform MVCs [45]. Further, a single test leader performed the experiments, and the study was performed in the settings of our laboratory. Therefore, the results cannot be generalized to other test leaders and other settings. Moreover, we acknowledge that the results cannot be extrapolated to other groups such as females or people with chronic pain. However, we believe that sEMG recordings can be performed longitudinally in workplace research and can be used to evaluate the effects of interventions aiming at reducing musculoskeletal load [11, 14].

## Conclusion

This reliability study showed that maximum absRMS and normRMS were found to have a fair to substantial relative inter-day reliability for most lifts but were more reliable when lifting from table to table than from floor to table both for trapezius descendens and erector spinae muscles. The relative inter-day reliability was higher for absolute compared with normalized sEMG amplitudes while the absolute reliability was similar. In addition, normRMS was more reliable for the erector spinae muscles than for the trapezius descendens muscles.

## Abbreviations

absRMS: Absolute root mean square
ICC_3,K_: Intraclass correlation
MDC: Minimal detectable change
MVC: Maximal voluntary contraction
normRMS: Normalized root mean square
SD: Standard deviation
SEM: Standard error of measurement
sEMG: Surface electromyography
